# Human salivary concentrations of brain derived neurotrophic factor correlates with subjective pain intensity associated with initial orthodontic therapy

**DOI:** 10.1038/s41598-023-28466-7

**Published:** 2023-01-31

**Authors:** Sagar S. Bhat, Ameet Vaman Revankar, Roopak D. Naik

**Affiliations:** 1 Department of Orthodontics, SDM College of Dental Sciences and Hospital, A Constituent Unit of Shri Dharmasthala Manjunatheshwara University, Dharwad, Karnataka 580009 India; 2 Department of Orthodontics and Dentofacial Orthopaedics, SDM College of Dental Sciences and Hospital, A Constituent Unit of Shri Dharmasthala Manjunatheshwara University, Dharwad, Karnataka 580009 India

**Keywords:** Biomarkers, Health care, Medical research, Molecular medicine, Signs and symptoms

## Abstract

Current study aimed to evaluate presence & concentration of salivary molecular pain biomarkers Calcitonin Gene Related Peptide (CGRP) and Brain-Derived Neurotrophic Factor (BDNF) during initial stages of orthodontic treatment and correlation with subjective pain scales, Numerical Rating Scale (NRS), Visual Analogue Scale (VAS), Verbal Rating Scale (VRS) and McGill Pain Questionnaire (MPQ). Consented, healthy-pain free patients (n = 40) undergoing orthodontic therapy, having moderate crowding with pre-molar extraction were recruited. Unstimulated whole saliva was collected and stored at -80 °C in cryotubes. Levels of CGRP & BDNF in salivary samples was assessed by enzyme–linked immunosorbent assay. Samples were collected under stipulated 5 time periods using saliva collection tube by passive drooling method: immediately after bonding but before wire placement (T0-baseline), after 24 h (T1), 48 h (T2), 72 h (T3) & 168 h (T4) after wire placement. Consolidated subjective pain scales were administered concurrently. Regression value (R^2^ > 0.9) confirmed BDNF & CGRP in saliva. Significant change was observed from baseline to 168 h in all subjective parameters (*p* < 0.05). CGRP did not correlate with subjective pain scales statistically (*p* > 0.05). BDNF levels correlated with all the subjective pain scales, NRS (T3-p = 0.0092&T4-p = 0.0064), VRS (T3-p = 0.0112&T4-p = 0.0500), VAS (T3-p = 0.0092 &T4-p = 0.0064) &MPQ (T1-p = 0.0255). Mean BDNF & median subjective pain scale graphs were similar. BDNF correlated with all the subjective pain scales warranting further investigation.

*Trial registration*; Clinical Trial Registry—India (CTRI) Reg No: CTRI/2018/12/016571; Registered 10th December, 2018 (10/12/2018) prospectively; http://ctri.nic.in/Clinicaltrials/pmaindet2.php?trialid=29640&EncHid=&userName=Dr%20Sagar%20S%20Bhat.

## Introduction

The pain experience is complex and fundamental to human existence, as reflected by its definition: “a sensory, unpleasant and emotional experience, associated with potential or actual tissue damage”^1^. Pain sensation is the most common, unpleasant experience perceived by orthodontic patients, that is induced by noxious stimuli due to inflammatory responses which are affected by several factors such as age, emotional status, gender and stress level of patients^2^. In orthodontic practice, pain may occur after the initial archwire placement immediately after bonding the brackets to the teeth, during the active phase of treatment, or post treatment^3^.

During orthodontic force application, the immediate and delayed painful responses were described by Burstone in 1962^4^. It was detected that there was hyperalgesia of periodontal ligament (PDL) leading to increased PDL sensitivity to noxious agents such as Prostaglandin E, Substance P (SP), and histamine which in turn lower pain threshold and also due to an initial compression of the PDL. Compression of PDL eventually leads to ischemia, inflammation, and later edema due to orthodontic force application^5^.

Painful perceptions are induced by activation of the orthodontic appliance, due to inflammatory process which occur as a part of tooth movement related to tissue remodeling. Previous evidences affirm that in the transmission of nociceptive information that were expressed bilaterally in the lateral parabrachial nucleus and ipsilaterally in the trigeminal subnucleus caudalis past the initial 24 h of orthodontic force application was due to the involvement of immunoreactive neuron C-fos. Similarly, fos-like immunoreactive neurons were distributed in other regions of brain such as the thalamic nucleus, neocortex, and dorsal raphe^6^. Nociceptive information by tooth movement is modulated and transmitted in several regions of the brain. Endogenous pain control systems are activated by these stimuli, including descending monoaminergic pathways^7^.

Preliminary studies established that dopaminergic and serotonergic systems regulate nociception^6^. Subsequently, another experiment performed showed an increase in serotonin turnover in the medulla, indicating the activation of bulbospinal serotonergic pathway by the nociceptive neurological response^7^. Consequently, an indirect nociceptive mechanism operates during tooth movement, suggesting a delayed and continuous nociceptive response, which regulates the masticatory function during active tooth movement^7^.

Conventionally, the extent of pain perception is assessed subjectively using several pain scales^1,8^. Over the years various subjective methods have been developed to assess pain more precisely, which include pain scales like NRS, VAS, VRS and MPQ^8^. The traditional simple descriptive pain scales, namely the visual analogue and graphic rating scales are more standardized and routinely used^8^. These pain scales are patient-friendly in being simple and easily understandable by the patients^8^. The use of these scales is the best available method for measuring pain or pain relief subjectively. Since there are subjective variations in pain with its duration and severity in an individual, objective assessment of pain is essential during orthodontic treatment^1^. Therefore, currently, the 'gold-standard' pain assessment tools rely on self-reporting, requiring an individual both to process external information and to communicate personal experience utilizing different subjective pain scales and questionnaires^9^.

Assessing pain objectively using salivary physiological biomarkers would benefit the clinician for appropriate pain diagnosis and management^1,3^. Even though well accepted by patients, saliva is frequently ignored as a body fluid of prognostic and diagnostic value due to the absence of a standardized collection procedure^10^. Bonetti et al. suggested that the fixed orthodontic appliance placement did not change the properties of saliva whilst assessed even one year after treatment when compared with the baseline^11^.

Objective biomarkers are defined as quantifiable characteristics of biological processes^10^. Nowadays many biomarkers are detected using saliva as a medium. It was recently discovered that several new isoforms for CGRP, BDNF, and Nerve Growth Factor (NGF) were found in saliva^10^. These were identified to develop new sensitive methods to analyze and detect biomarkers related to pain.

NGF is a neuropeptide functioning as a protective component for neurons facilitating neuronal regeneration and plays an important role in hyperalgesia. Its concentration increases during inflammation which is amplified in response to noxious stimuli^12^. BDNF and CGRP plays a major role in the development of hyperalgesia and pain. They have been involved in headache and migraine based on increased saliva and plasma concentrations during active pain periods^12,13^. Few of the studies have investigated the levels of these above-mentioned neuropeptides in saliva^12–14^.

A variety of findings regarding the duration of pain are reported in studies associated with pain in orthodontic treatment. Several patients describe much longer periods of pain and discomfort which are common during the first 1 or 2 days of the orthodontic treatment. Scheurer et al.^15^ reported that even after 7 days of initiation of a fixed appliance, 25% of all investigated patients still reported pain^15^. According to measurements at 4 h and 24 h, the pain intensity generally increases with time but falls to normal levels after 7 days of initiation of orthodontic treatment^15,16^.

This is a novel study correlating CGRP and BDNF with subjective pain scales in pain experienced with initial orthodontic therapy. Therefore, the current study aims to evaluate the presence and concentration of CGRP and BDNF in whole unstimulated saliva and to correlate these with subjective pain scales VAS, VRS, NRS, and MPQ during initial alignment stage of fixed orthodontic treatment in subjects with moderate crowding.

## Materials and methods

Patients reporting to the Department of Orthodontics and Dentofacial Orthopaedics, SDM College of Dental Sciences and Hospital Dharwad were included in this study after approval from the Institutional Ethics Committee (IEC)—IRB Number: 2018/P/ OR/55 on 15/11/2018. This single arm, prospective study was registered prospectively at Indian Council of Medical Research (ICMR)—Clinical Trial Registry India (CTRI) Reg No: CTRI/2018/12/016571 on 10/12/2018.

### Research participants and study design

**Figure 1:**
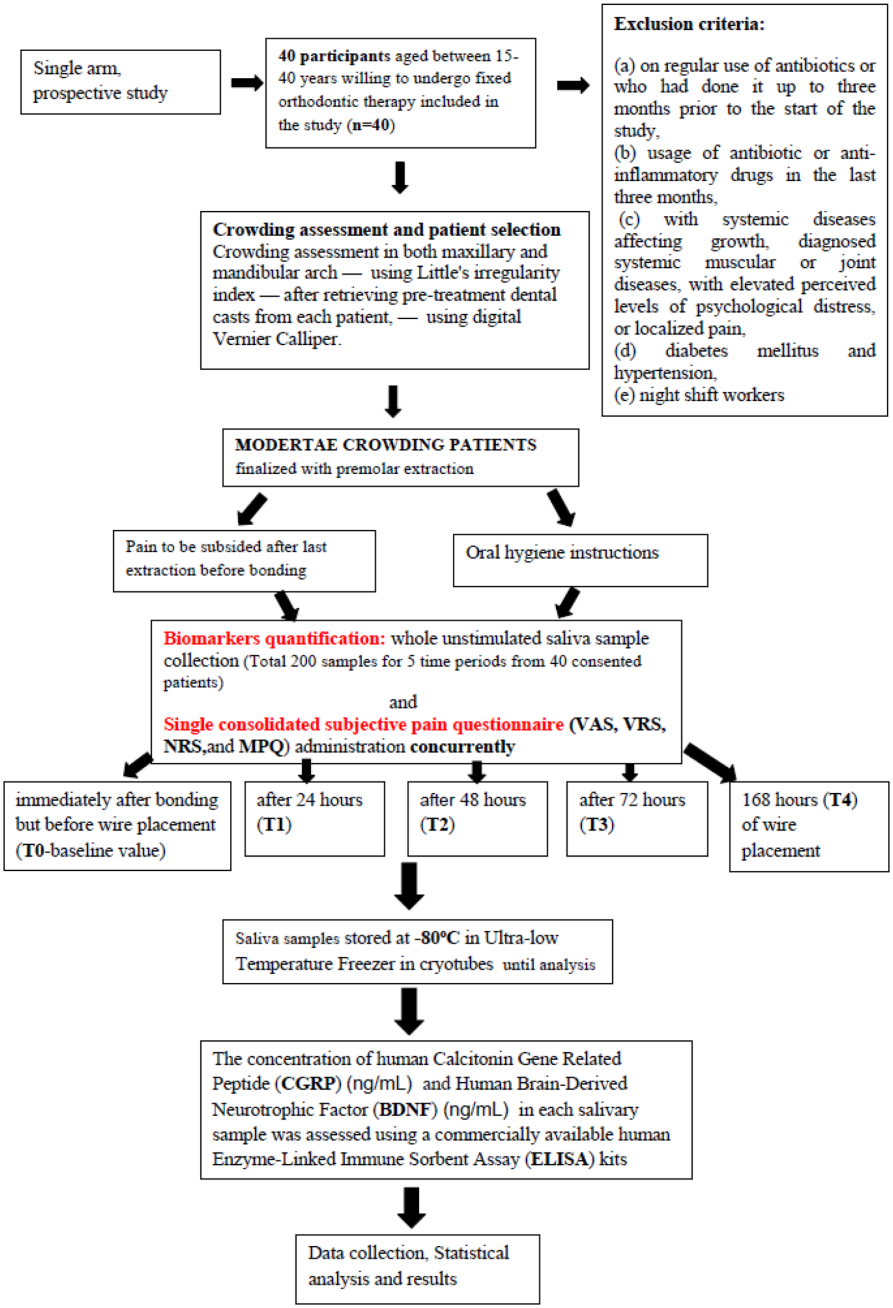
Flowchart of the study.

This study was designed with a sample size of 40 subjects (32 females, 8 males). Healthy pain—free subjects with good oral hygiene, free of fever/ cold, aged between 15 and 40 years with a mean age of 19.4 years were included in the study after obtaining their informed consent. And for patients below age group of 16, the informed consent was obtained from their respective parents/guardians. Patients with systemic diseases affecting growth, diagnosed systemic muscular or joint diseases, with elevated perceived levels of psychological distress, localized pain, night-shift work or under any medications were excluded from the study. No specific tests were conducted in this regard. It was orally discussed with the patient and patient information was obtained by means of an interview. A flowchart with the study design is shown in Fig. 1.

### Clinical examination, crowding assessment and patient selection

Fixed mechanotherapy using Pre-Adjusted Edgewise brackets with MBT prescription (3 M Gemini, 3 M Unitek Corporation, California) having 0.022 × 0.028″ slot was commenced following the maxillary and mandibular first or second bicuspid extraction. Leveling and alignment of the arches was done on a 0.014″ round NiTi archwire (Optima, Orthodontic Supplies Ltd, Leicestershire, UK).

The patient was bonded one week following the last extraction after the pain from extraction had subsided. To assess the amount of crowding in both maxillary and mandibular arch, Little's irregularity index in the maxilla and mandible was used after retrieving pre-treatment dental casts from each patient which was measured using digital Vernier Calliper—Absolute digimatic (Mitutoyo Corporation—Takatsu Ward, Kawasaki, Kanagawa, Japan) and the crowding severity was graded. Selected patients were in the grade ‘moderate’ crowding. In the maxillary arch, the variables that were assessed are the maxillary irregularity index, arch length, inter-canine, inter-premolar and intermolar widths.

### Biomarkers quantification: Saliva sampling

Unstimulated whole saliva samples were collected in test tubes for each patient. It was stored at − 80 °C in Ultra-low Temperature Freezer (− 80 °C—New Brunswick Scientific *Co* Inc. Freshwater Blvd Enfield, CT, USA) in cryotubes—Abdos Freezing Tubes PP, Sterile − 80 °C—P60103—(ABDOS Lifesciences—ABDOS Labtech Private Limited—Jasola, New Delhi, India)**—**until analysis and the concentration of human Calcitonin Gene Related Peptide (CGRP—Cat No: K12—1062) (ng/mL) and Human Brain-Derived Neurotrophic Factor (BDNF—Cat No: K12—1303) (ng/mL) in each salivary sample was assessed using commercially available human Enzyme-Linked Immune Sorbent Assay (ELISA) kits (KINESISDx—Krishgen Biosystems—Los Angeles, CA, USA).

All the subjects participating in the study were instructed not to drink, eat or brush their teeth 1 h prior to the saliva collection, and not consume any alcoholic beverages 24 h prior to collection. They were also instructed to avoid dietary products rich in tryptophan content like red meat, eggs, fish, nuts, seeds and yoghurt and to maintain a detailed food log 24 h prior to collection. The subjects were reinforced with oral hygiene instructions during the initial appliance placement before commencing the study. All the patients complied to the oral hygiene regimen of oral prophylaxis followed by rinsing twice a day with Chlorhexidine gluconate (0.2%) and brushing twice daily was also advised, all through the course of the study.

Samples were collected under stipulated 5 time periods using saliva collection tube by the passive drooling method: immediately after bonding but before wire placement (T0-baseline value), after 24 h (T1), 48 h (T2), 72 h (T3), and 168 h (T4) of wire placement, giving a total of 200 samples i.e.; 40 subjects, 5 samples from each subject.

### Subjective questionnaire design

A single consolidated questionnaire constituting the patient's basic demographic details, condensed case history, and following pain scales: VAS, VRS, NRS, and MPQ was administered to each subject concurrently.

### Statistical analysis

The sample size was estimated using GraphPad Prism, Statistical Package for the Social Sciences (SPSS) Version 17 software. Considering the effect size ie; the incidence population to be measured (ρ) at 20% i.e.; correlation coefficient between the variables at 0.30, incidence of study group at 40%, power of the study (1-β error) at 80%, β error is 0.2 and the margin of the error (α) at 0.05%, the total sample size was estimated to 36. Anticipating 17% attrition during the follow-up, the total sample size was rounded off to 40.

Data was analysed using statistical software SPSS version 20.0 software (IBM, Armonk, New York, USA) and Microsoft Excel (Microsoft Corporation, One Microsoft Way Redmond, Washington, U.S.A). There were 40 subjects in the sample. Categorical variables are given in the form of frequency table. Continuous variables are given in mean ± SD/median (range) form. To compare mean/distribution within the time points, one-way repeated measures of analysis of variance (ANOVA)/Friedman’s test, and Spearman Rank correlation coefficient was used for data analysis. Regression analysis was performed. Analysis of ELISA optical density (OD) was done using 4—parametric logistic regression curve analysis to use these as clinical biomarkers. Correlation coefficient analysis among and between pain scales was performed. The level of significance was set at 5% (*p* < 0.05).

### Ethical approval

All procedures performed in studies involving human participants were in accordance with the ethical standards of the institutional research/review board (IRB)- IRB Number: 2018/P/ OR/55 and with the 1964 Helsinki Declaration and its later amendments or comparable ethical standards.

### Declaration of patient consent

The authors certify that they have obtained all appropriate patient consent. The patient has given his/her consent for his/her images and other clinical information to be reported in the journal. And the patient understands that their names and initials will not be published and due efforts will be made to conceal their identity, but anonymity cannot be guaranteed. For patients below age group of 16, the consent was obtained from their respective parents/guardians.

### Informed consent statement

Informed consent was obtained from all the subjects who participated in this study. And for patients below age group of 16, the informed consent was obtained from their respective parents/guardians.

## Results

**Figure 2:**
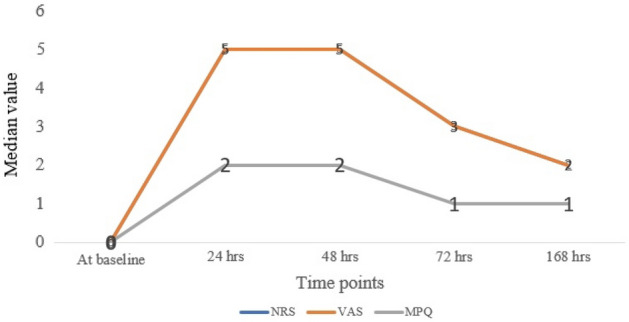
Median of Numerical Rating Scale (NRS), Visual Analogue Scale (VAS), and McGill Pain Questionnaire (MPQ) over time points.

A total of 40 participants were considered for the study with mean age group of 19.4 years + 3.04 months. All the patients were subjected to subjective pain scoring, as shown in Table 1. In all the subjective pain scoring there was a significant (*p* < 0.0001) change over time. The median values of the subjective score were plotted as shown in Fig. 2. It shows that pain scores were high at 24 h and subsided after 48 h, heading towards the baseline. Median values of all subjective pain score except VRS was displayed over time points. In the graph, NRS and VAS lines are merged. According to the 4-parametric logistic regression curve with the BDNF standards, the test was performing well (R^2^ > 0.9). Regression analysis (4-parametric analysis) with the commercially available cytokine kit with BDNF and CGRP was satisfactory with an R^2^ of 0.97 and 0.95 respectively (Figs. 5, 6). The dose–response curve was plotted simultaneously.

**Table 1:** Comparison of subjective pain score namely VAS, VRS, NRS and MPQ over time points.

Variable	Time points					*p*-value
	At baseline	At 24 h	At 48 h	At 72 h	At 168 h	
NRS	0 (0, 0)	5 (1, 9)	5 (0, 8)	3 (0, 9)	2 (0, 7)	< 0.0001*
VRS						
None	40 (100%)	0 (0%)	12 (30%)	3 (7.5%)	9 (22.5%)	< 0.0001*
Mild	0 (0%)	5 (12.5%)	17 (42.5%)	22 (55%)	25 (62.5%)	
Moderate	0 (0%)	23 (57.5%)	1 (2.5%)	12 (30%)	5 (12.5%)	
Severe	0 (0%)	12 (30%)	10 (25%)	3 (7.5%)	1 (2.5%)	
VAS	0 (0, 0)	5 (1, 9)	5 (0, 8)	3 (0, 9)	2 (0, 7)	< 0.0001*
MPQ	0 (0, 0)	2 (1, 3)	2 (0, 3)	1 (0, 3)	1 (0, 3)	< 0.0001*

Significant values are in [bold]. *p < 0.05.

**Figure 3:**
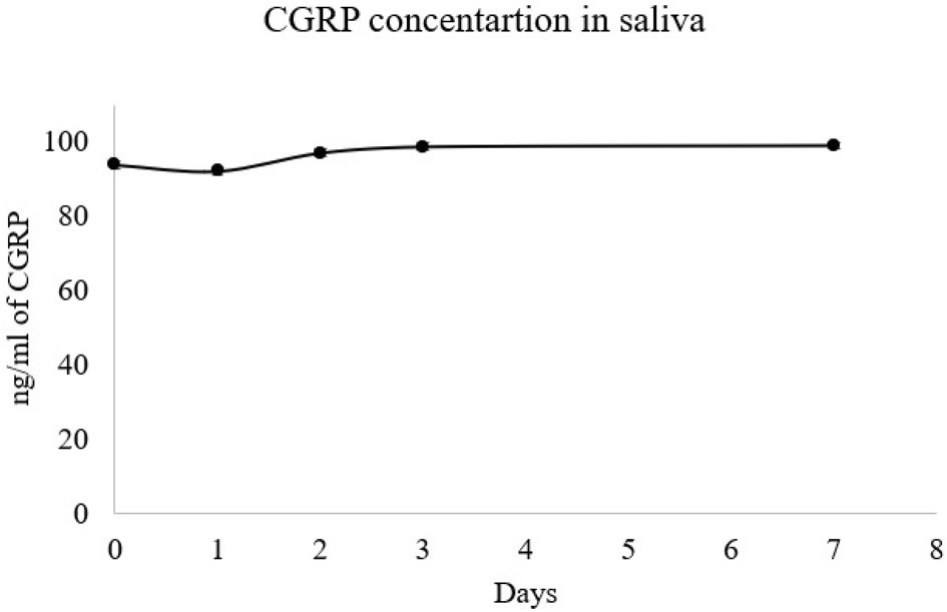
CGRP concentration among the patients at different time interval.

**Figure 4:**
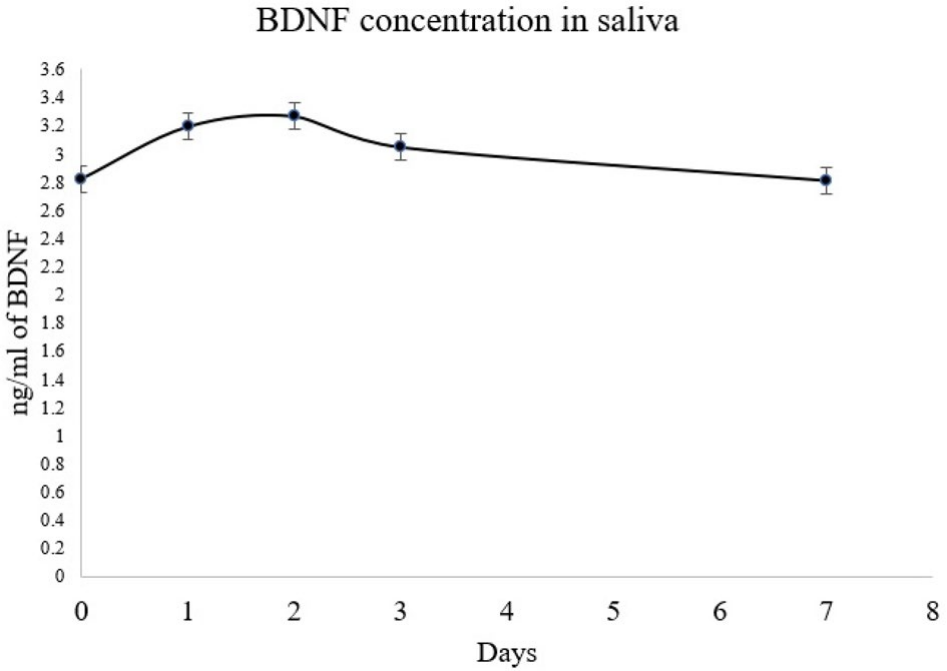
BDNF concentration among the patients at different time interval.

Timeline study of CGRP showed that on 2nd day there was a slight decline in the CGRP levels but subsequently it went on increasing till day 7 (Fig. 3). Upon comparison of the CGRP levels at each time point with the baseline, there was no significant change in the values. There was no difference between CGRP between baseline and at each time point. Timeline study of BDNF showed that it was increasing till 3rd day further started declining till day 7 (Fig. 4). Upon comparison of the BDNF levels at each time point with the baseline, there was no significant change.

**Figure 5:**
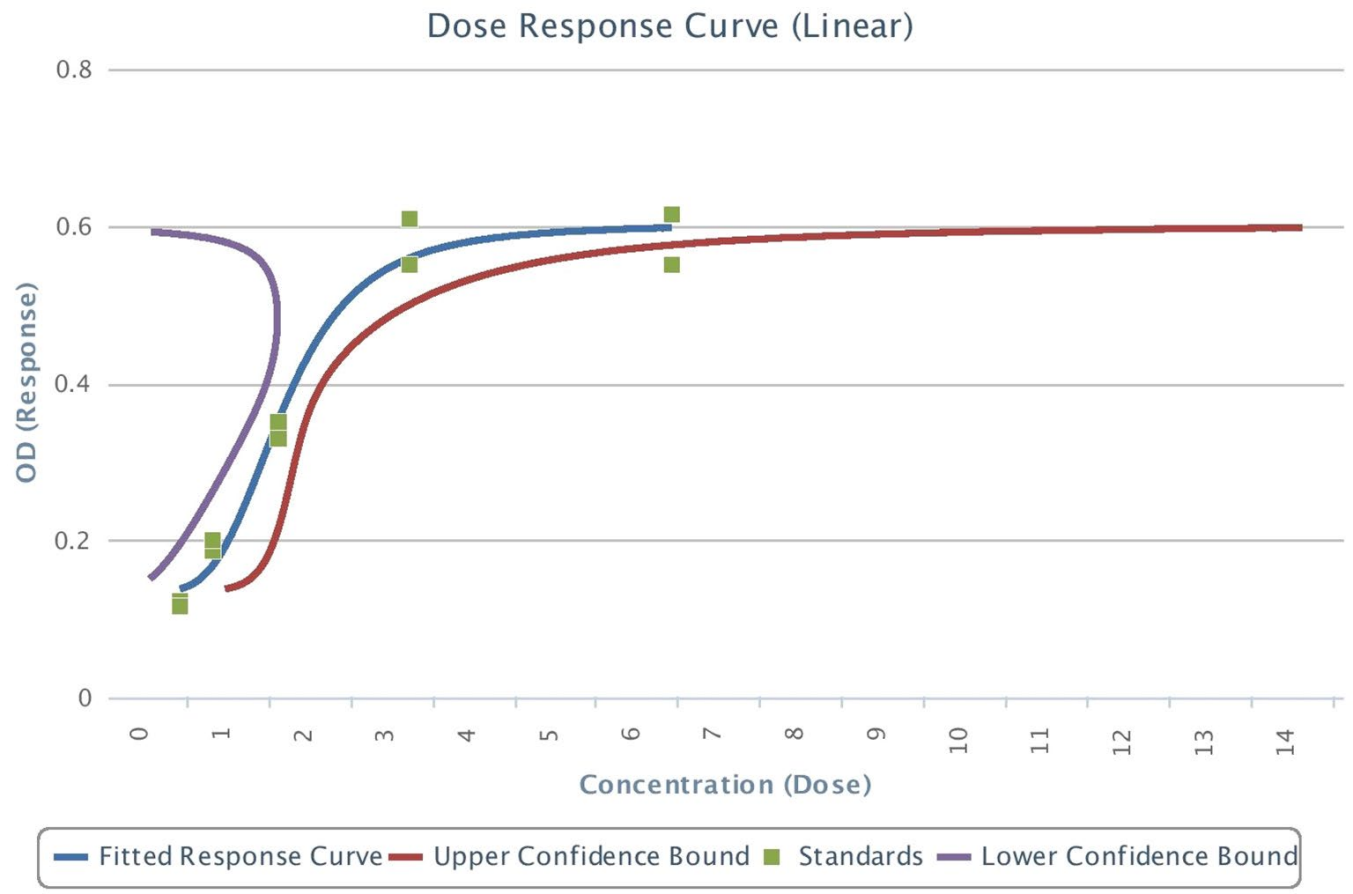
BDNF analysis by 4-parametric logistic regression curve analysis with commercially available standards.

**Figure 6:**
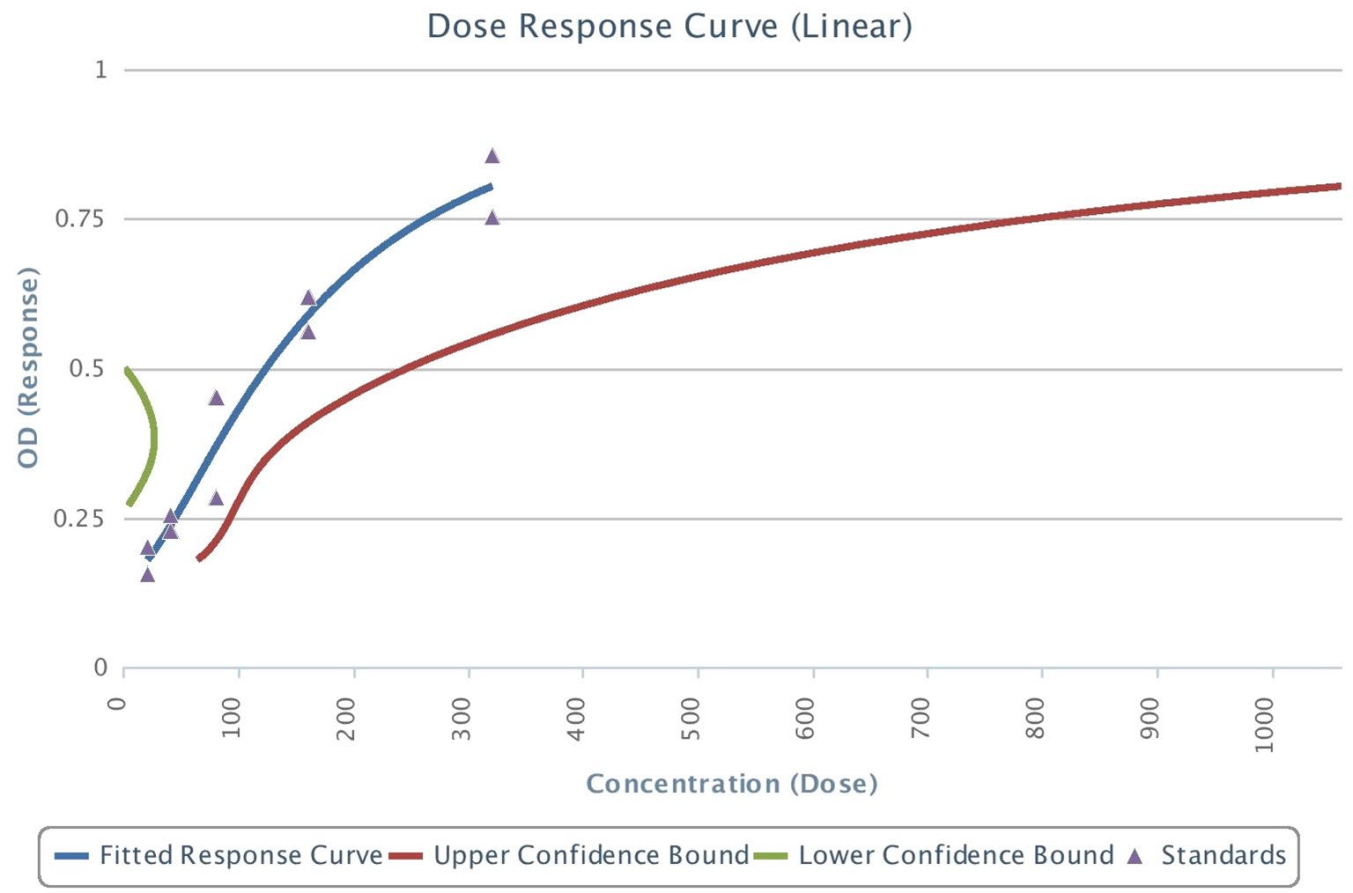
CGRP analysis by 4-parametric logistic regression curve analysis with commercially available standards.

Table 2 also shows overall CGRP and BDNF change over the timeline. Further to correlate between subjective scores and objective levels, correlation between CGRP and BDNF with NRS, VRS, VAS and MPQ scores by Spearman’s rank correlation was performed respectively (Tables 3, 4). Change of CGRP at different time points are negatively correlated but not statistically significant with the subjective scales (VAS, VRS, NRS and MPQ) changes at different time points (*p* > 0.05). Change of BDNF at different time points are negatively correlated and is statistically significant with the subjective scales at T3 (72 h) and T4 (168 h) of NRS (T3-p = 0.0092&T4-p = 0.0064), VRS (T3-p = 0.0112&T4-p = 0.0500), VAS (T3-p = 0.0092 &T4-p = 0.0064) & MPQ (T1-p = 0.0255). When the subjective scores were compared among each other over that time point, there was a significant difference in the distribution of VAS and MPQ, NRS and MPQ over all time points as shown in Table 5 (*p* < 0.0001). Comparison among objective results was made over the time point, it showed that there was a significant difference between CGRP and BDNF as shown in Table 6 (*p* < 0.05) (Figs. 5, 6).

**Table 2:** Comparison of objective pain score – molecular pain biomarker CGRP and BDNF over time points.

	Time points					*p*-value
	At baseline	At 24 h	At 48 h	At 72 h	At 168 h	
CGRP						
Mean (sd)	92.1 ± 44.34	86.67 ± 49.69	96.22 ± 64.14	97.39 ± 58.02	98.77 ± 68.5	0.9913
Median (Range)	96.05 (13.2, 208.543)	84.2 (0, 228.2)	85.7 (0, 279.9435)	96.95 (0, 244.2)	98.8 (0, 317.9)	
BDNF						
Mean (sd)	2.84 ± 2.04	3.17 ± 1.95	3.27 ± 1.53	3.06 ± 1.7	2.8 ± 1.99	0.39
Median (Range)	3.1 (0, 8.9)	3.299 (0, 10)	3.4 (0, 7.4)	3.4 (0, 6.6)	2.95 (0, 9.8)

**Table 3:** Correlation between Calcitonin Gene Related Peptide (CGRP) with NRS, VRS, VAS and MPQ scores by Spearman’s rank correlation.

Variables	Time points	T1		T2		T3		T4	
		Spearman R	*p*-value	Spearman R	*p*-value	Spearman R	*p*-value	Spearman R	*p*-value
NRS	T0	–	–	–	–	–	–	–	–
	T1	− 0.1447	0.3731						
	T2			− 0.0203	0.9013				
	T3					− 0.1519	0.3495		
	T4							− 0.1845	0.2545
VRS	T0	–	–	–	–	–	–	–	–
	T1	− 0.1553	0.3388						
	T2			0.0143	0.9302				
	T3					− 0.0759	0.6415		
	T4							− 0.2079	0.1980
VAS	T0	–	–	–	–	–	–	–	–
	T1	− 0.1447	0.3731						
	T2			− 0.0203	0.9013				
	T3					− 0.1519	0.3495		
	T4							− 0.1845	0.2545
MPQ	T0	–	–	–	–	–	–	–	–
	T1	− 0.2657	0.0976						
	T2			0.0022	0.9891				
	T3					− 0.0691	0.6719		
	T4							− 0.1918	0.2359

**Table 4:** Correlation between Brain Derived Neurotrophic Factor (BDNF) with NRS, VRS, VAS and MPQ scores by Spearman’s rank correlation.

Variables	Time points	T1		T2		T3		T4	
		Spearman R	*p*-value	Spearman R	*p*-value	Spearman R	*p*-value	Spearman R	*p*-value
NRS	T0	–	–	–	–	–	–	–	–
	T1	− 0.2096	0.1943						
	T2			− 0.2560	0.1108				
	T3					− 0.4066	0.0092*		
	T4							− 0.4241	0.0064*
VRS	T0	–	–	–	–	–	–	–	–
	T1	− 0.2095	0.1944						
	T2			− 0.2080	0.1977				
	T3					− 0.3972	0.0112*		
	T4							− 0.3038	0.0500*
VAS	T0	–	–	–	–	–	–	–	–
	T1	− 0.2096	0.1943						
	T2			− 0.2560	0.1108				
	T3					− 0.4066	0.0092*		
	T4							− 0.4241	0.0064*
MPQ	T0	–	–	–	–	–	–	–	–
	T1	− 0.3530	0.0255*						
	T2			− 0.1358	0.4033				
	T3					− 0.1964	0.2244		
	T4							− 0.2624	0.1019

Significant values are in [bold].

*p < 0.05.

**Table 5:** *p*-values of Comparison between subjective pain score – VAS, NRS and MPQ over time points.

Subjective pain score	Time points				
	At baseline	At 24 h	At 48 h	At 72 h	At 168 h
NRS					
VAS	–	1	1	1	1
MPQ	–	< 0.0001*	< 0.0001*	< 0.0001*	0.007928*
VAS					
MPQ	–	< 0.0001*	< 0.0001*	< 0.0001*	0.007928*

Significant values are in [bold]. *p < 0.05.

**Table 6:** *p*-values of comparison between objective pain score – molecular salivary pain biomarkers namely CGRP and BDNF over time points.

	Objective pain score	Time points			
	At baseline	At 24 h	At 48 h	At 72 h	At 168 h
CGRP					
BDNF	–	< 0.0001*	< 0.0001*	< 0.0001*	0.007928*

Significant values are in [bold]. *p < 0.05.

## Discussion

Pain accompanies fixed orthodontic treatment. The intensity of pain perception during fixed orthodontic tooth movement is often described by its physiology and various evaluation methods which are specifically and generally associated with the amount and duration of an orthodontic force application.

With the advancements that have taken place over the last few decades in the field of analytical technologies for saliva, it has gained an intensified attention for clinical and laboratory diagnostics. Biomarkers of saliva as a measure have the potential to be an objective approach and a diagnostic tool for the studies related to pain. However, there is a need of estimating the different collection methods and develop improved techniques for analysis. These biomarkers have a crucial part in objectively assessing the pain.

Salivary α -amylase activity was assessed by Campos et al^2^ to reveal any correlation between the salivary levels of α-amylase and pain intensity reported by patients during orthodontic treatment. The study findings concluded that analysis of α-amylase concentrations was not sufficient to determine the pain objectively experienced by patients undergoing fixed orthodontic treatment. Salivary α-amylase levels did not show a significant relationship with reported pain intensity. Nevertheless, the progressive increase in salivary α -amylase concentration was observed during the study period signifying an emotional stress overload in these patients, as a response at the start of treatment. In this study, for objective pain measurements BDNF and CGRP were evaluated. Timeline study of CGRP showed that 2nd day there was a slight decline in the CGRP levels but subsequently it went on increasing till day 7. Timeline study of BDNF showed that it was increasing till 3rd day and started declining till day 7. There was a significant difference between objective pain scores over time points. Therefore, BDNF is proposed as a better objective pain biomarker in assessing pain associated with orthodontic therapy.

Increased expression of SP and CGRP during the first two days after orthodontic force application in rats^17,18^ was found to be appealing, considering the findings clinically by conducting human studies who elicit pain perception reaching peak approximately one to two days after force application leads to expression of the same markers^19–22^. A research study was conducted by Silva et al^23^ to evaluate the levels and concentration of stress biomarkers and electrolytes in saliva of patients undergoing fixed orthodontic treatment. The emotional stress level of the orthodontic patients was higher than the controls only on the day after appliance activation, as detected by the α-amylase activity. The significant increase in pain during chewing 24 h after activation of the appliance was significantly correlated with a decrease in the masticatory performance of patients. In our study, further to correlate between subjective scores and objective levels, there was significant difference between objective pain score namely CGRP and BDNF over time points (*p* < 0.0001). CGRP did not show statistical correlation (*p* > 0.05) with any of the subjective pain scales. Therefore, pain experienced by the subject may not be objectively assessed by CGRP. BDNF levels correlated with all the subjective pain scales, NRS (at T3 – p = 0.0092 and T4 – p = 0.0064), VRS (at T3 – p = 0.0112 and T4 – p = 0.0500), VAS (at T3 – p = 0.0092 and T4 – p = 0.0064) and MPQ (at T1 – p = 0.0255). Furthermore, the graph of mean BDNF concentrations showed a similar pattern with median values of subjective pain scales NRS, VAS and MPQ plotted over all time points. There was validation of the presence of both salivary physiological markers BDNF and CGRP during the fixed orthodontic therapy. There was a more significant distribution of BDNF concentration when compared with subjective pain scores with contrast to CGRP concentration. From our study results, it was elicited that BDNF correlated partially with pain scales at different intervals.

Till date, in the research study of any field there are no validated objective pain markers. Only few validated subjective pain scales like VAS, VRS, NRS and MPQ are used to assess the amount of pain perception the patient is experiencing during any treatment. So, this study can be a small eye opener which provides a valuable insight to perform more research in discovering many more objective pain markers which can differentiate between pain experience in patients. This study is the first of its kind.

In absence of clear knowledge regarding the aforementioned molecular biomarkers, we are just at the tip of the iceberg and the scope for future research in the subject is vast and challenging. The individual response to BDNF and CGRP in the field of orthodontic treatment due to orthodontic force application may be variable and this may lead to find out if there are any differences in pain experience while using different prescription or bracket type in future. Also, studies to estimate the amount of pain perception in different stages of fixed orthodontic treatment and genetic variations of the BDNF and CGRP expression should be carried out to determine if there are any differences in the expression of these cytokines in different populations. Additional research in this field using BDNF and CGRP concentration in saliva can help in the development of an objective pain scale for assessing pain experience chairside, which will be more reliable and validated.

## Conclusions

CGRP did not show statistical correlation (*p* > 0.05) with any of the subjective pain scales. BDNF levels showed statistically significant (*p* < 0.05) correlation with VAS, VRS, NRS at T3 and T4 and MPQ at T1 time intervals. Although at other intervals there was a correlation (Spearman R < 0), it was statistically insignificant (*p* > 0.05). Furthermore, the graph of mean BDNF concentrations showed a similar pattern with median values of subjective pain scales NRS, VAS and MPQ plotted over all time points. BDNF correlated with all the subjective pain scales warranting further investigation.

### Clinical significance

Clinicians would be benefitted by obtaining an objective scale for assessing pain, since many patients are unable to exactly differentiate between the pain or pressure. Also, this would be very useful in treating special needs patients who are generally unable to express themselves. Research based on introducing this objective tool in daily clinical practice will help to optimize the pain perception in patients during different stages of fixed orthodontic therapy.

### Shortcomings of the study

The severity of the malocclusion in each patient can also play a role in BDNF and CGRP levels that were produced. Only moderate crowding patients were employed in this study. Further studies in mild and severe crowding patients with different malocclusions may be necessary to obtain a better picture. A myriad of environmental factors and the inherited genetic traits have been long proven to alter pain perception to orthodontic force application during fixed orthodontic treatment. Hence, these should have been accounted for in the current study. Age-related changes and how they manifest in each patient can also affect the outcome of the results, as pain might be due to many psychological, sociocultural, emotional and stress related factors. In children, the effect of puberty and fluctuations of hormonal levels could also account in variations in the expression of BDNF and CGRP. More research is required in this field, as pain is a very vast discipline.

## Data Availability

The datasets used and/or analysed during the current study are available from the corresponding author on reasonable request.
